# Conversations and Medical News Frames on Twitter: Infodemiological Study on COVID-19 in South Korea

**DOI:** 10.2196/18897

**Published:** 2020-05-05

**Authors:** Han Woo Park, Sejung Park, Miyoung Chong

**Affiliations:** 1 Department of Media & Communication Interdisciplinary Graduate Programs of Digital Convergence Business and East Asian Cultural Studies Yeungnam University Gyeongsan-si Republic of Korea; 2 Cyber Emotions Research Institute Gyeongsan-si Republic of Korea; 3 Tim Russert Department of Communication John Carroll University Cleveland Heights, OH United States; 4 College of Information University of North Texas Denton, TX United States

**Keywords:** infodemiology, COVID-19, SARS-CoV-2, coronavirus, Twitter, South Korea, medical news, social media, pandemic, outbreak, infectious disease, public health

## Abstract

**Background:**

SARS-CoV-2 (severe acute respiratory coronavirus 2) was spreading rapidly in South Korea at the end of February 2020 following its initial outbreak in China, making Korea the new center of global attention. The role of social media amid the current coronavirus disease (COVID-19) pandemic has often been criticized, but little systematic research has been conducted on this issue. Social media functions as a convenient source of information in pandemic situations.

**Objective:**

Few infodemiology studies have applied network analysis in conjunction with content analysis. This study investigates information transmission networks and news-sharing behaviors regarding COVID-19 on Twitter in Korea. The real time aggregation of social media data can serve as a starting point for designing strategic messages for health campaigns and establishing an effective communication system during this outbreak.

**Methods:**

Korean COVID-19-related Twitter data were collected on February 29, 2020. Our final sample comprised of 43,832 users and 78,233 relationships on Twitter. We generated four networks in terms of key issues regarding COVID-19 in Korea. This study comparatively investigates how COVID-19-related issues have circulated on Twitter through network analysis. Next, we classified top news channels shared via tweets. Lastly, we conducted a content analysis of news frames used in the top-shared sources.

**Results:**

The network analysis suggests that the spread of information was faster in the Coronavirus network than in the other networks (Corona19, Shincheon, and Daegu). People who used the word “Coronavirus” communicated more frequently with each other. The spread of information was faster, and the diameter value was lower than for those who used other terms. Many of the news items highlighted the positive roles being played by individuals and groups, directing readers’ attention to the crisis. Ethical issues such as deviant behavior among the population and an entertainment frame highlighting celebrity donations also emerged often. There was a significant difference in the use of nonportal (n=14) and portal news (n=26) sites between the four network types. The news frames used in the top sources were similar across the networks (*P*=.89, 95% CI 0.004-0.006). Tweets containing medically framed news articles (mean 7.571, SD 1.988) were found to be more popular than tweets that included news articles adopting nonmedical frames (mean 5.060, SD 2.904; N=40, *P*=.03, 95% CI 0.169-4.852).

**Conclusions:**

Most of the popular news on Twitter had nonmedical frames. Nevertheless, the spillover effect of the news articles that delivered medical information about COVID-19 was greater than that of news with nonmedical frames. Social media network analytics cannot replace the work of public health officials; however, monitoring public conversations and media news that propagates rapidly can assist public health professionals in their complex and fast-paced decision-making processes.

## Introduction

### Background

SARS-CoV-2 (severe acute respiratory coronavirus 2) is spreading rapidly around the world, and the number of associated deaths has also been increasing. At the end of February 2020, the virus was spreading in South Korea following its initial outbreak in China, making Korea the new center of global attention. Mass infection occurred in Korea due to a closed religious group called *Shincheonji* in the greater Daegu metropolitan city, the fourth largest city in Korea [1,2].

Social media has been criticized often amid the current coronavirus disease (COVID-19) pandemic, mainly due to their use as a medium for the quick spread of fake news [3,4], but no systematic research has yet been conducted on this issue. Social media functions as a convenient source of information in dangerous situations [5]. Since the creation of social networking services (SNS) such as Facebook, Twitter, and YouTube, the speed of information transmission in disaster contexts has accelerated across social, cultural, and geographical boundaries. Real time information exchange through various SNS can facilitate the wider diffusion of risk information not only for “friends” but also for wider communities.

Using an infodemiological approach, this study analyzes networking trends in public conversations and news-sharing behavior regarding COVID-19, particularly in Daegu, South Korea, on Twitter. The Pew Research Center reported that approximately 75% of Twitter users visit Twitter.com to read the news [6]. Allowed up to 280 characters, Twitter users share thoughts and emotions through “tweets” and “retweets,” which creates conversational and networked relationships on Twitter. The pattern of interactions between Twitter users may vary according to their interests and engagement with COVID-19. This study examines conversations on Twitter in relation to the greater Daegu metropolitan city cluster that was closely related to the members of the Shincheonji group, who contributed significantly to spreading the virus in the area. Four Twitter networks were chosen—Coronavirus, Corona19, Daegu, and Shincheonji—to represent the major issues regarding the COVID-19 crisis in the greater Daegu area. The keyword “Corona19” was included instead of “COVID-19” (coronavirus disease) because “Corona19” was announced as the official term for COVID-19 in Korea.

The Twitterverse examined in this context includes diverse messages on topics such as nationwide emergency relief efforts, media news, mass condolences, requests for central and regional governmental measures, and the provision of crucial medical information. The fact that these four networks have similar network sizes allows their conversational patterns and news diffusion to be easily compared.

Overcoming the current COVID-19 crisis may require increasingly diverse forms of data and more complex models. Handling the real time aggregation and artificial intelligence-based analytics of social media, media news, academic publications, and other data sets is a daunting task. Nevertheless, this study could serve as a starting point for designing strategic messages for health campaigns and establishing an effective communication channel system.

### Infodemiology

Infodemiology is a growing area of research that aims to inform public health officials and develop public policies using informatics for the analysis of health data produced and consumed online [7]. The advantage of infodemiology is its capacity to obtain real time health-related data from unstructured, textual, image, or user-generated information communicated via electronic media such as websites, blogs, and social network sites [8].

Infodemiology studies have covered a wide range of topics. These include information search behaviors such as Ebola- or vaccination-related information [9,10], health-related news coverage [11], public health issues and awareness of diseases after the death of a celebrity [10], disparities in health information access and availability [12], public discussion and information sharing [13], and government risk communication strategies [14].

The trustworthiness of user-created information is questionable [15]. However, recent studies suggest that publicly available social media data such as those on Twitter and Facebook can complement traditional epidemiologic data and methods such as hospital- or pharmacy-based data, clinical data, focus interviews, and surveys, which are time-consuming [16,17].

In particular, user-generated content and shared health information on social media can serve as an alternative tool for syndromic surveillance [8,18]. Social media health data can accelerate data collection, curation, and analysis. Analyzing user content (especially on Twitter) and tracking information usage patterns such as users’ browsing, searching, clicking, or sharing of information regarding health care can reflect the health status, concerns, awareness, and health-related behaviors of the public [18-22]. User-generated content and shared information on social media can also be used for knowledge translation and to increase awareness among policymakers [8].

Investigating the public’s communication framing of and approaches to health issues as observed on social media provides insights into the public’s thoughts on, perceptions about, and self-disclosures of disease-related symptoms [13]. This can ultimately assist in the development of health intervention strategies and the design of effective campaigns based on public perceptions.

Studies have focused on quantifying the search queries and tracking the volume of health-related information or user-generated content in electronic media using Google Trends, Google Health application programming interface (API), or Google Flu Trends [23,24]. Recent studies in the fields of risk communication have applied social network analysis to investigate the structure of knowledge and information-sharing networks and multilevel interaction patterns among users [14,25,26]. Studies suggest that network analysis is particularly useful for tracking not only collaborative networks among different stakeholders but also the necessary source distribution during national disasters or emergencies such as earthquakes [14,25,26]. The timely monitoring of risk networks and public conversations on social media can help foster an understanding of stakeholders’ perspectives and assist in establishing the policies required for effective risk interventions and resilience, thus, ensuring the effective management of catastrophic events [27-29].

### Objectives

We address three research questions (RQs) about Korea’s COVID-19 conversations in terms of socially disseminated Twitter messages. First, is there a difference in communication network structure among the four networks generated from four keywords (written in Korean)—Coronavirus, Corona19, Shincheonji, and Daegu—and what are the characteristics of the conversation patterns among users? Second, which news topics and media channels generate the users’ interest, and what are their characteristics? Do the most frequently mentioned news topics among the four networks display any differences in media outlet type? Third, what perspectives on news articles are observed from a media organizational point of view? In other words, do news articles with a medically oriented thematic frame have broader spillover effects on the COVID-19 issue in the Twitter context?

## Methods

### Data Collection

This study evaluates trends in Korea’s COVID-19 conversations using Twitter data. Data were collected on February 29, 2020, roughly covering the most recent weeks in the Twitter database. Using the Twitter search API embedded in NodeXL (Social Media Research Foundation) [30], we crawled Twitter users whose recent tweets contained the term “Coronavirus” (코로나바이러스), “corona19” (코로나19), “Shincheonji” (신천지), or “Daegu” (대구) in Korean. These four terms represent the key issues related to COVID-19 in Korea. The tweets were taken from a data set limited to a maximum of around 18,000 tweets. Twitter is one of the largest social media services in the world and provides conversational data, regardless of personal information collection and use consent, that are available free of charge via various automatic methods. Our final sample comprises 43,832 Twitter users and 78,233 relationships, which includes “tweets,” “retweets,” “replies,” and “mentions” from the selected Twitter networks. Once Twitter users post tweets, which can include text, hashtags, images, and URLs, the entire content of the tweets is included when they are retweeted or mentioned by other Twitter users [31].

### Social Media Network Analysis and News Channel Classification

This study used three main methodological approaches. It conducted a social media network analysis to determine how COVID-19-related issues circulate on Twitter. The study traced the characteristics of information diffusion regarding COVID-19 on Twitter by generating communication networks composed of all tweets containing any of the search terms (“Coronavirus,” “corona19,” “Shincheonji,” or “Daegu”). A communication network in this study refers to a social network generated by users to communicate to each other. Each node represents a user, and the links between the users refer to a conversation (ie, a retweet, reply to, or mention). Four networks were generated. A network analysis was conducted to identify the multidimensional communication activities between Twitter users and grasp the nature of the information transmission networks composed of entities such as words, hyperlinks, and hashtags. Twitter users are compiled by subgroup using the Clauset–Newman–Moore cluster algorithm and visualized using the Harel–Koren Fast Multiscale layout algorithm [32]. This method has been widely used in communication research on information measurement [33,34]. The study uses NodeXL to calculate various measures quantifying the efficiency of information diffusion and visualizing the structural topography of the network associated with the results of the four search queries as seeds. The network measures include modularity, the number of self-loops, and connected components. Modularity is an indicator of the community structure [35]. The higher the modularity value, the greater the subgroup interconnection. Components in a network analysis refers to a subgraph that represents nodes connected by paths [36]. A self-loop occurs when virtually no one replies to or mentions a tweet.

We then classified the top news items in terms of their media channels. The media outlet of a news article can be regarded as both a form of carrier interface and a means of expression. In Korea, portals are increasingly becoming the primary point of news access. Given Korea’s unique news environment, a media organization’s presence on a portal offers an interface between its news production and its readership [37]. Furthermore, a professional news agency provides a means of diffusing breaking news quickly and effectively. Media outlets were classified as portal or nonportal news. News stories that were delivered in Korean information portals such as Naver, Daum, and Yahoo! were categorized as portal news. News stories that were not provided through information portals were considered nonportal news. Intercoder reliability scores were calculated based on the coding of 20% (n=8/40) of the sample by a second independent coder using Cohen kappa. There was a strong agreement between the two coders (κ=0.71, *P*=.03).

### Content Analysis of News Frames Used in COVID-19 News Coverage

Besides news channels, we also considered news frames as points of view that guide readers’ cognitive direction. Content analysis was conducted to determine the main frames within the news stories by generating content categories that encompass the entire text corpus [38]. News frames were categorized into medical or nonmedical frames. A total of 40 popular news items across the four networks were categorized as “medical vs nonmedical” in terms of their frames. When a news story covered a medical or health issue related to COVID-19, it was classified as a “medical frame.”

A coding scheme was developed to further subclassify the nonmedical news frames based on studies of journalists’ use of news frames [39,40] and on an inductive review of the news contained in the tweets. The coding scheme was designed to capture how the newspapers promote a specific definition, interpretation, or evaluation of social issues [41]. Nonmedical news frames were classified into five categories: “attribution of responsibility,” “human interest,” “morality,” “entertainment,” and “conflict” [39,40]. Textbox 1 presents the definitions of each frame. When more than one frame was used, the dominant frame was selected. Each news item is classified as one frame by one coder (one of the authors). Intercoder reliability scores were computed based on the coding of 20% (n=8/40) of the sample by a second coder using the kappa coefficient. There was a very strong agreement between the two coders (κ=0.837, *P*<.001).

**Definitions of news frames used in coronavirus disease news coverage.**

**Conflict**

Reflects disagreement between parties, individuals, or groups

**Human interest**

Emphasizes the (positive) role of individuals and groups affecting the issue

**Attribution of responsibility**

Suggests that some government agencies, including politicians and public officials, are responsible for the issue

**Morality**

Contains a moral and ethical message

**Medical**

Mentions medical and health issues related to the problem

**Entertainment**

Covers cultural issues such as celebrity, sports, or food

## Results

### Comparing Communication Network Structures

**Figure 1:**
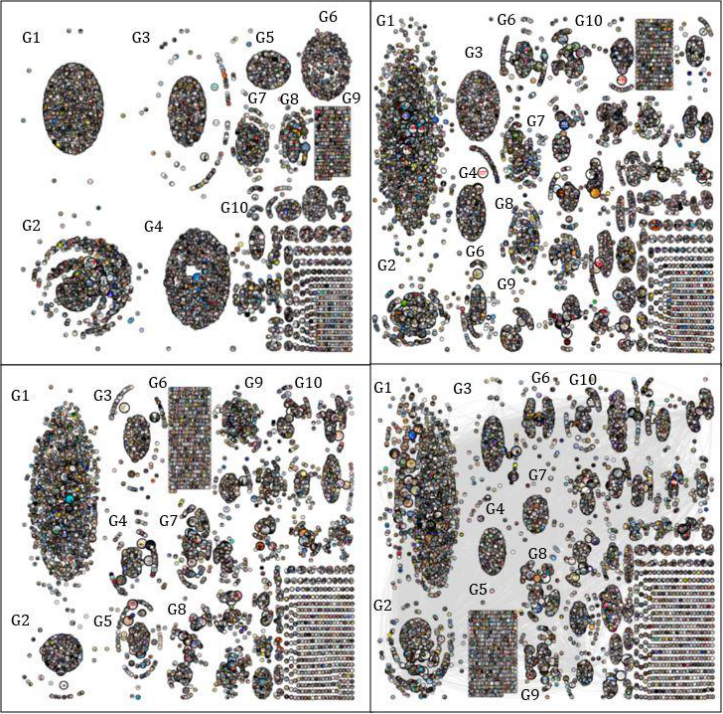
Korean coronavirus disease networks on Twitter. Coronavirus (top left), Corona19 (top right), Shincheonji (bottom left), and Daegu (bottom right).

We addressed RQ1 by comparing the topologies of the four networks, which are shown in Figure 1. The top 10 subgroups are indicated in the graphs. In undirected networks, the size of the radius of the nodes reflects the size of their betweenness centrality values. Due to space constraints, we did not present the ties between nodes.

As Figure 1 shows, the patterns of conversation and information sharing between users are similar across all networks. The results suggest a complex structure composed of multiple layers. Around 20 major subcommunities were activated, and many interactions occurred at an individual level with a small number of isolated users. Table 1 outlines the characteristics of the conversational relationships among users in each network.

The frequency of unique edges was lowest in the Coronavirus network. Unique edges reflect frequency, excluding redundant relationships. In other words, the Coronavirus network has the most redundant relationships, indicating that people continued to talk to each other while exchanging comments several times. Thus, it is highly likely that a “big mouth” existed in the Coronavirus network. On the other hand, the Daegu and Corona19 networks had the lowest frequencies of edges with duplicates. These fewer overlapping relationships suggest that many one-time conversations took place, forming an instant and improvised community.

There are many self-loops in the Shincheonji network, wherein tweets started and ended with the same user in a conversation thread. The Shincheonji network has the highest reciprocated vertex pair and reciprocated edge ratios, which shows that “birds of a feather flock together.” When two Twitter users talk to each other, their relationship is regarded as being reciprocated. In sharp contrast, less than one-tenth of tweets in the Coronavirus network were self-loops, revealing that different types of users were paired in comment exchanges. Similarly, the Shincheonji network also had the largest number of isolates, followed by the Daegu network. An isolate has zero connections. This result suggests that the communication patterns in region-oriented networks, Shincheonji and Daegu, differed from the networks that concerned more overall issues regarding COVID-19. The difference can be attributed to geographic variation in information-sharing behaviors on Twitter [42].

**Table 1:** Comparing user relationships across coronavirus disease networks.

Network measures	Coronavirus	Corona19	Shincheonji	Daegu
Nodes, n	12,803	11,739	9589	9701
Isolates, n (%)	368 (2.87)	324 (2.76)	471 (4.91)	434 (4.47)
Total edges, n	18,407	19,772	20,327	19,727
Unique edges, n (%)	14,486 (78.70)	17,042 (86.19)	16,879 (83.04)	17,017 (86.26)
Edges with duplicates, n (%)	3921 (21.30)	2730 (13.81)	3448 (16.96)	2710 (13.74)
Self-loops, n (%)	1450 (7.88)	2318 (11.72)	2754 (13.55)	1901 (9.64)
Reciprocated vertex pair ratio	0.00020	0.00042	0.00353	0.00334
Reciprocated edge ratio	0.00040	0.00084	0.00704	0.00666

Table 2 summarizes the overall attributes of each network. When a Twitter network is drawn in concentric form, the step required to connect two users, potentially through intermediate users, reflects the “geodesic” value. The path that connects the furthest pair is the maximum geodesic distance, or the diameter of the network. The Coronavirus network has the smallest diameter and average values among the four networks. Because people who used the word “Coronavirus” communicated much more frequently with each other than those who used other terms, the spread of information was much faster, and thus, its value is the lowest. By contrast, the Corona19 network has the largest geodesic values.

Next, the modularity value of the Coronavirus network was the highest among the four networks, while the Daegu network had the lowest value. This result suggests that the clusters created within the Coronavirus network may be less cohesive in terms of the subgroups’ internal collectivity because the Twitter users in group A tend to be connected with other users in group B. If modularity is low, the clusters are well-defined in terms of the quality of the subgroups generated. Because the Daegu and Shincheonji networks both have lower modularity than the other networks, users classified in the same cluster rarely left their own group to talk to others in a different cluster.

A component analysis reveals that Shincheonji and Daegu network members had the largest numbers of connected components and the maximum edges in a connected component. Coronavirus network members had the largest chat room with the highest value for maximum vertices (ie, users) in a connected component, followed by the users of Corona19.

**Table 2:** Comparing coronavirus disease network properties on Twitter.

Types	Coronavirus	Corona19	Shincheonji	Daegu
Maximum geodesic distance (diameter)	12	16	14	14
Average geodesic distance	3.865	5.459	4.432	4.471
Modularity	0.674	0.667	0.563	0.530
Connected components, n	584	582	799	828
Maximum vertices in a connected component, n	10,783	10,135	8270	8098
Maximum edges in a connected component, n	16,121	17,916	18,947	18,123

### Popular News Topics and Frequently Cited Media Outlets

RQ2 addresses the popularity of news topics and media channels. We extracted the most cited news among the four networks. Five news items appeared twice on the top 10 list of the four Twitter networks. The most popular news concerned suspicions that the *Daily Best*’s online bulletin board deleted all the posts that mentioned Shincheonji. The second most popular news was about the warning given by the Korea Centers for Disease Control that 18 types of beards could be dangerous amid the COVID-19 risk. The third most popular news was the breaking news that the COVID-19 infection rate among ordinary citizens was low in Daegu, unlike for members of Shincheonji. The fourth-ranked news item concerned how messages of support from all over the country, including handwritten letters and fruit, gave Daegu medical staff the strength to continue working. The fifth-ranked concerned news was that college students had created a fact-checking site to prevent the spread of fake news related to COVID-19.

The top 10 news stories on the Twitterverse related to Shincheonji included the term “Shincheonji” in their headline titles. For example, the most popular news was that Shincheonji leader Lee Man-hee had received recognition for his service to the country from ex-President Park Geun-hye and that he was set to be buried at the National Cemetery. Thus, the top news stories shared on Twitter featured eye-catching headlines, the use of dramatic expressions, and emotional narratives.

It is noteworthy that international news, rather than domestic news, was chosen as the top news item. *Foreign Policy* (FP), a US diplomatic magazine, diagnosed the cause of Korea’s COVID-19 problem [43]. The article was titled “Cults and Conservatives Spread Coronavirus in South Korea: Seoul Seemed to Have the Virus under Control, but Religion and Politics Have Derailed Plans.” Additionally, a petition posted to the presidential “Blue House” was also a top news item [44]. The petition called for the dismissal of Prosecutor General Yoon Seok-Yeol for failure of leadership because he did not deal promptly with the Shincheonji-related situation despite the deep concerns of the Korean public.

Finally, we investigated the most cited news channels among the four networks. Korea’s portals and *Yonhap News* (Korea’s largest news agency, comparable to the US Associated Press) were ranked highly in frequently linked Twitter groups (n=26 and n=8, respectively). The preferred channel of popular news in the Coronavirus Twitter network was *Yonhap News*. Among the portals, *Daum*, the second largest portal in Korea, was overwhelmingly more popular than *Naver*, the largest portal in Korea, (n=22 and n=4, respectively). The rest include online-focused newspapers (n=2), social media postings (n=2, with one tweet pointing to the FP article and the other citing the famous “Real-time in Daegu” Facebook page), a personal blog (n=1), and a petition site (n=1). The dominance of the portals and *Yonap News* is attributable to their business model of delivering news faster than other media outlets do. Surprisingly, no traditional media outlet appears among the top news channels.

The study computed a 4 (Corona19 vs Coronavirus vs Daegu vs Shincheonji) x 2 (portal vs nonportals) chi-square comparing the frequency of portal vs nonportal news site use between network types. The difference is found to be significant (χ^2^_3_=12.747, *P*=.005). The results indicated that the Corona 19 network cited more portal news (n=8/10, 80%) than did nonportal news (n=2, 20%), whereas the Coronavirus network used more nonportal sources (n=8/10, 80%) than portal news (n=2, 20%), *P*=.007. The Coronavirus network also used more nonportal sources than portal sources, while the Daegu network (*P*=.002) and Shincheonji network (*P*=.02) included more portal news (n=9/10, 90% for Daegu; n=7/10, 70% for Shincheonji) than nonportal sources (n=1, 10% for Daegu; n=3, 30% for Shincheonji).

We also determined the origin of the news items reported in the portals. The four *Naver* news articles were based on *SEN*, the *Sports Donga* newspaper (both online-focused newspapers), *Newsis* (a news agency), and *Maeil Broadcasting Network* (a cable news channel, comparable to CNN). Some 22 *Daum* news articles came from the *News1* (news agency; n=3); *Newsis* (n=2); *OhMyNews*, an online-focused newspaper (n=1); *Nocut* News, an online-focused newspaper (n=4); the *Seoul Daily* newspaper (n=2); the *World Daily* newspaper (n=1); *Yonhap News* (n=4); the *JoongAng Daily* newspaper (n=2); the *Hankyoreh Daily* newspaper (n=2); and the *Hankook Daily* newspaper (n=1). The so-called “legacy” media are eager to provide their news via Korea’s portals because that enables their breaking and exclusive news articles to attract greater attention from the portals’ readership.

### News Frames and Popularity of News

This study addresses RQ3 by analyzing the news frames of media organizations that were circulated in tweets. The results of content analysis show that 17.5% (n=7/40) of the articles mentioned medical or health problems. The medical news items include discussions of the characteristics of COVID-19, warnings about the potential for beards to be infected with COVID-19, the effects of health conditions on mortality, the status of COVID-19 tests in Italy, and the difference in infection rates between Shincheonji members and ordinary citizens.

The results of an independent two-tailed *t*-test suggest that tweets containing medically framed news articles (mean 7.571, SD 1.988) are found to be more popular than tweets that included news articles adopting nonmedical frames (mean 5.060, SD 2.904, *P*=.03, 95% CI 0.169-4.852).

This study ranked the most popular news articles from 1 to 10. This study then calculated reverse scores to measure the spillover effects of the articles. For example, the top-ranked news item were given 10 points, and the 10th-ranked item was given 1 point.

Lastly, the study investigated the news frames included in the tweets produced across the four networks and compared the association between the network typology and the frames. The findings suggest that the “attribution of responsibility” frame was the most frequently used, followed by “human interest.” Both “morality” and “entertainment” were cited 6 times. “Conflict” was the least used frame. This study conducted a 4 (network types) x 6 (news frames) chi-square analysis to examine the association between network type and the news frames used in the tweets. As shown in Table 3, the results indicate that network type was not significantly associated with the news frames (*P*=.89, 95% CI 0.004-0.006), suggesting that the six frames were used similarly across the four networks.

**Table 3:** Chi-square results for the news frames across coronavirus disease networks.

Network type		Total (N=40), n (%)	Conflict (n=4), n (%)	Entertainment (n=6), n (%)	Human interest (n=7), n (%)	Medical (n=7), n (%)	Morality (n=6), n (%)	Attribution of responsibility (n=11), n (%)	Chi-square (*df*)	
**All networks**		N/A^a^	N/A	N/A	N/A	N/A	N/A	N/A		8.727 (15)
	Corona19	10 (25)	1 (10)^b^	2 (20)	2 (20)	3 (30)	1 (10)	1 (10)		N/A
	Coronavirus	10 (25)	0 (0)	1 (10)	2 (20)	2 (20)	2 (20)	3 (30)		N/A
	Daegu	10 (25)	0 (0)	2 (20)	2 (20)	1 (10)	1 (10)	4 (40)		N/A
	Shincheonji	10 (25)	2 (20)	1 (10)	1 (10)	1 (10)	2 (20)	3 (30)		N/A

^a^Not applicable.

^b^In the calculation of the n (%) values across each row, the row total is taken as the N value.

## Discussion

### Principal Findings

By March 1, 2020, Korea had become one of the most SARS-CoV-2-infected countries in the world. The greater Daegu metropolitan area had Korea’s highest COVID-19 infection rate per household as well as the highest absolute rate [45]. This study has found that the spread of information was faster in the Coronavirus network than in the other networks because Twitter users in cluster A created within that network tended to be interconnected with others in cluster B. These findings have implications for risk communication and health campaigns. When government authorities and experts share and comment on real time information about ongoing infectious disease threats, social media analytics can help them to choose what keywords and hashtags are more appropriate to use. In fact, central and regional governments in Korea have been criticized for causing social confusion by failing to effectively communicate proper information on how to deal with the symptoms of infectious diseases. Our findings can be used to enhance government fact-checking services and risk communication. For example, the frequently shared topics and information sources regarding the Shincheonji-related Twitter networks could help both local and central governments to review people’s concerns and opinions regarding the case and to guide information strategies in epidemic situations, which often demand prompt decision making for risk management.

Our main research platform was Twitter, but the analysis has also considered intermedia journalism that goes beyond Twitter. People use various news channels to share information, even when communicating via social media. This study found that portals were the preferred news sources on Twitter. As shown in Endo’s [46] study on Japan’s 3.11 earthquake, various media interact with each other during national disasters, creating a social information environment that can be both positive and negative. It would be ideal if intermedia journalism created an information immunization system during epidemics. This study is important in that it examines a key aspect of intermedia journalism; although, the full dynamics of intermedia journalism during Korea’s COVID-19 crisis has yet to be fully investigated.

### Limitations

Although this study proposed and demonstrated a useful infodemiology framework by performing social network analytics to explore information diffusion related to the COVID-19 pandemic via Twitter in Korea, it is not without limitations. These limitations are inherent in Twitter’s user population. The literature suggests that only 15% of online adults are regular Twitter users [47]. Moreover, the largest group among Twitter users is composed of those 18-29 years of age. In addition, only a small number of Twitter users are active in producing tweets and leveraging the discourse [47-49]. There are more very passive (<50 tweets per year) and very active users (>1000 tweets per year) on Twitter than moderate users (50-1000 tweets a year) [46]. Thus, the study’s results may reflect social media users’ views and behaviors during the pandemic rather than the full population’s aggregate opinion. In addition, biases in information-sharing behaviors can exist, as some users may have produced more content than others. It was also possible that whether they are residents or visitors of Daegu City may have influenced what news they shared [50]. Furthermore, we qualitatively examined the study’s data sets to detect abnormal activities such as data contamination from social bots; however, running Twitter bot detection software would have enabled us to more systematically remove any potential biases from the data set. Finally, we applied multi-coder methods, but two coders may not be sufficient to ensure reliability. Future studies could apply more coders to guarantee the reliability of the analysis results.

### Conclusions

People experiencing social disasters such as epidemics of infectious diseases are unfamiliar with their situation and find it difficult to predict what will happen next. Therefore, risk communication that delivers accurate and appropriate information is important. We found that most of the popular news on Korea’s Twitterverse had nonmedical frames. Nevertheless, it must be noted that the spillover effect of the news articles that delivered medical information about COVID-19 was greater than that of news with nonmedical frames. For instance, many news items reported that the initial response of government agencies was responsible for the spread of COVID-19. Many news items highlighted the positive role of individuals and groups, directing readers’ attention to the epidemic crisis. Ethical issues such as deviant behavior among the population and an entertainment frame highlighting celebrity donations also emerged often. Relatively few articles reflected discrepancies in positions or opinions among individuals or groups.

Augmented intelligence systems in the medical sector have been widely cited as an important approach to helping detect and clinically diagnose diseases [51]. However, this study is not intended to provide public health care data to artificial intelligence systems, such as HealthMap or BlueDot, to assist in the prediction of infectious disease occurrence. Rather, this study emphasizes the need and opportunity for strategic communication through social media. Social media network analytics cannot replace the work of public health officials; however, collecting public conversations and media news that propagates rapidly and detecting its structure can assist public health professionals in their complex and fast-paced decision-making processes.

## Abbreviations

API: application programming interface
COVID-19: coronavirus disease
FP: Foreign Policy
RQ: research question
SARS-CoV-2: severe acute respiratory coronavirus 2
SNS: social networking services
